# Resilience and Subjectively Experienced Stress Among Paramedics Prior to and During the COVID-19 Pandemic

**DOI:** 10.3389/fpsyg.2021.664540

**Published:** 2021-07-15

**Authors:** Andrzej Piotrowski, Ryszard Makarowski, Radu Predoiu, Alexandra Predoiu, Ole Boe

**Affiliations:** ^1^Faculty of Social Sciences, Institute of Psychology, University of Gdańsk, Gdańsk, Poland; ^2^Faculty of Health Sciences, Elbląg University of Humanities and Economics, Elblag, Poland; ^3^Teachers’ Training Department, National University of Physical Education and Sports, Bucharest, Romania; ^4^Sports and Motor Performance Department, Faculty of Physical Education and Sport, National University of Physical Education and Sports, Bucharest, Romania; ^5^USN School of Business, Department of Industrial Economics, Strategy and Political Science, University of South-Eastern Norway, Drammen, Norway

**Keywords:** stress, resilience, personal resources, paramedics, pandemic, COVID-19

## Abstract

**Introduction:**

Paramedics play a vital role in the healthcare system by providing professional support in situations of direct threat to patient health and life. They experience numerous difficulties during their work, which result in occupational stress. During the COVID-19 pandemic, their work has become even more demanding. The aim of the current study was to examine the role of resilience in the subjective experience of stress among paramedics during the COVID-19 pandemic.

**Materials and methods:**

The study was carried out in two phases, in October-November 2019 (*N* = 75) and in May-June 2020 (*N* = 84), using the Sense of Stress Questionnaire (*Skala Poczucia Stresu*) and the Resilience Scale (*Skala Pomiaru Prężności*).

**Results:**

Paramedics exhibited higher *intrapsychic stress* before the COVID-19 pandemic. *Tolerance of failure and treating life as a challenge* were higher during the pandemic, in contrast to *optimism and the ability to mobilize in difficult situations*. Paramedics who were in contact with patients with COVID-19 experienced higher stress. *Perseverance and determination, openness to new experiences and sense of humor*, as well as *competences* and *tolerance of negative emotions* were revealed to play a key part in mitigating subjectively experienced stress.

**Conclusion:**

Paramedics’ subjectively experienced stress was lower during the COVID-19 pandemic. Paramedics who were in direct contact with patients with COVID-19 experienced higher stress. They had sufficient psychological resources, in the form of resilience (*perseverance and determination, openness to new experiences, sense of humor*, and *competences and tolerance of negative emotions*), which allowed them to cope with the situation of the COVID-19 pandemic.

## Introduction

In March 2020, the World Health Organization (WHO) announced COVID-19 to be a global pandemic. Together with the governments of nearly all countries in the world, the WHO decided that coordinated strategies are required in order to limit its spread. These strategies differed between the countries. However, the corona virus pandemic (COVID-19) has negatively impacted nearly each sector of global society regardless, and the healthcare sector has borne the brunt of this burden, unprecedented for the last hundred years, the most. The COVID-19 that recently hit the entire world has revealed a previously unseen number of consequences for physical and mental health of individuals and for society at large (Hanna et al., 2018; Brooks et al., 2020; Fiorillo and Gorwood, 2020; Lima et al., 2020). Working on the first line of contact with patients in situations of sudden health or life risk, paramedics play an important role in the healthcare system. This role has become even more important during the COVID-19 pandemic due to the increased number of people requiring immediate medical attention. The work of a paramedic involves high physical demands, such as being routinely exposed to physical demands, such as lifting, lowering, carrying, pushing, and pulling (Coffey et al., 2016). They are also exposed to high psychological demands, and their well-being is further impacted by stress and traumatic experiences (Ogińska-Bulik, 2014a).

Due to the character of their work, paramedics are at risk for workplace accidents (Garus-Pakowska et al., 2016). Moreover, their work involves traumatic experiences, such as contact with death (Ogińska-Bulik, 2014a,b; Rasmus et al., 2020). During the COVID-19 pandemic, the healthcare system has become severely strained by the number of additional patients. Direct contact of the medical staff with bodily fluids and pathogens in the air puts them at an especially high risk of infection with the SARS-CoV-2 coronavirus (Tysiąc-Miśta et al., 2021). Research results show that there is a risk of infection through contact with contaminated surfaces and items (Hanke and Pietrzak, 2021). In turn, due to unavoidable contact with the patients’ bodily fluids and airborne transmission, paramedics are at a high risk of exposure to pathogens (Gańczak and Topczewska, 2018; Tysiąc-Miśta and Bulanda, 2021).

The pandemic situation has caused concern for own life as well as the life of one’s significant others to become a constant source of stress for paramedics. Their work has also become more demanding due to an increased number of patients and a reduced number of available personnel due to infection and quarantine. In many countries, medical staff make up for the greatest amount of COVID-19 deaths—estimated at 30% in the initial stages of the pandemic (Nolan et al., 2020). Such constant sense of threat can cause intense stress in paramedics (Stasiła-Sieradzka and Turska, 2019). Directly witnessing the effects of the disease, awareness of the number of deaths, and difficulties in preventing and combating infections are sources of anxiety for paramedics (Shahzad et al., 2020). However, such anxiety is common, as research shows that around 80% of all Poles are afraid of contracting COVID-19, and over 85% experience related anxiety, restlessness, and tension (Babicki and Mastalerz-Migas, 2020).

### Stress at Work

Anxiety and stress at work can affect both well-being and sleep pattern of employees (in our case, paramedics). The work conditions in the job environment (task-related stressors, social stressors, role stressors), and the subjective experience of these conditions could cause affective, cognitive, and physiological reactions in paramedics. But, if some individuals/employees will experience work stress and might be affected, others might not (and not suffering any strain)—Carleton and Barling (2017). We can discuss “healthy stress” (stress at work which is prone to stimulate challenges) and “unhealthy stress”—work stress that leads to hardship, distress or strain, resulting in diminishing individual’s work well-being (Kanji and Chopra, 2009). An important element in reducing work stress is worktime control, which is negatively linked with distressing experiences in employees (Weiß, 2020). Also, crystallized and fluid cognitive abilities are valuable personal resources that protect workers against the negative effects of work-related stress—crystallized cognition plays a more important role for older than younger employees (Hyun et al., 2018). Not least, work stress could be reduced through developing programs/interventions for work-leisure conflict, knowing that such a variable (as a mediator) is significantly influenced by workaholic behaviors, and determines a high level of work stress (Meier et al., 2020).

Generally speaking, stress at work is significantly linked with sleep problems (Berset et al., 2011; Stenfors et al., 2013). In this context, it seems that interpersonal stress is more important than time related stressors and task-related stressors in affecting sleep quality (Nixon et al., 2011). Fear of losing a job or social approval may result in low levels of secondary traumatization reporting among healthcare providers (Greinacher et al., 2019). Exposure to psychologically traumatic events (PPTEs) can be an unavoidable occupational risk in these professions. Healthcare workers (911 call center operators/dispatchers, paramedics, doctors, nurses) are exposed to high stress in the workplace and the associated risk of mental disorders (Krakauer et al., 2020). Research suggests that nearly 45% of public safety personnel tested positive for at least one mental disorder (Carleton et al., 2019). Over 80% of paramedics reported having experienced an “extremely disturbing incident” in the past six months (Greinacher et al., 2019). Studies show consistent links between negative psychological outcomes and the safety and quality of healthcare (Johnson et al., 2017; Cleary et al., 2018).

Also, paramedic teams are regularly exposed to a variety of clinical incidents, including fatalities or unsuccessful resuscitation outcomes, and may even be victims of assault and verbal threats. All of these events can adversely affect the physical and mental well-being of paramedics. Additionally, there has been a profound transformation in delivery services, leading to increased personnel pressure to achieve goals (Clompus and Albarran, 2016). For example, in the United Kingdom, there is a nationally agreed standard for Category A (life-threatening) urgent calls requiring emergency vehicles to arrive at the scene within 8 min in 75% of the cases (HSCIC, 2014). Shift work patterns and single work have been changed, and the growing administrative workload may have a cumulative effect on the productivity and coping capacity of employees.

### Resilience

Personal resources, including resilience, are important in coping with stress. Researchers agree that resilience is a relatively constant personal disposition determining the process of flexible adaptation to constantly changing life demands (Ogińska-Bulik and Juczyński, 2008). Resilience is defined as an individual trait which aids effective coping with difficult and traumatic situations. Highly resilient people are productive, perseverant, and emotionally stable. They have a sense of humor, perceive situations mainly as challenges, and finish started tasks. High resilience aids in coherent and adequate adaptation to changing circumstances. It can be understood as a relatively constant personal resource which becomes manifest during significant life difficulties or threats. On the other hand, resilience can be understood through the process account, which refers to dynamic and positive adaptation in the face of difficulty (Luthar et al., 2000). Understood as a dynamic property, resilience depends on a specific life context, and thus, it can be developed (Ogińska-Bulik and Juczyński, 2008). Resilience can protect against negative effects of stress (Ogińska-Bulik and Michalska, 2019) and have a direct or indirect effect on post-traumatic functioning in paramedics (Ogińska-Bulik and Kobylarczyk, 2015). Resilience is not seen as an innate trait or as a stable or static individual trait, but it can be developed or destroyed in an unpredictable way and can be seen as a set of tools and strategies that a person builds when faced with difficult situations (Hunter and Warren, 2014). One of the goals of a study conducted by Gayton and Lovell (2012) was to assess whether the time of paramedic service is associated with increased resilience, or whether the paramedic profession simply attracts people with a high level of resilience. The results indicated that experienced paramedics showed a significantly higher level of resilience than the student paramedics. These findings bolster calls for increased resilience interventions for paramedics and paramedical students to protect their well-being.

The topic of employee resilience in the workplace is an area of increasing interest in the medical profession, but there is little research on it within the paramedical profession (Clompus and Albarran, 2016). The concept of resilience in the health sector was drawn from the literature on child development and focused on causal risk mechanisms and protective factors for workers (McAllister, 2007). However, the notion of resistance as an individual trait has been superseded by the work of Luthar et al. (2000), who understood it as a dynamic process in which internal (psychological) and external (social, e.g., gender, socioeconomic status) factors interact.

Health risks increase with insufficient number of paramedics. Some medical staff were unable to work during the COVID-19 pandemic due to their health condition or fear of losing it. Resilience is a psychological variable that allows to predict the readiness of paramedics to respond to situations that threaten their health (chemical, biological, radiological, nuclear, and explosives—CBRNE) (Stevens et al., 2010). Qureshi et al. (2005) argued that anxiety for oneself and family was a factor associated with the lowest willingness to work during disasters among health care workers who also rated biological and chemical incidents as types of events of greatest concern.

A meta-analysis of research by Mason et al. (2020) indicates that paramedics with higher neuroticism and low extraversion were more likely to experience critical incidents at work as personality traits could account for more than 30 percent of changes in resilience (Froutan et al., 2018). Additionally, a literature review conducted by Phillips (2019) indicates that the resilience of rescuers is influenced by the types of stressors they struggle with and the coping strategies used. Some coping strategies are modifiable, as confirmed by studies suggesting that not only can resilience be developed, but that resilience may also decrease over time.

A study by Gilbert (2018) revealed that previous experiences of traumatic incidents strengthened the participant’s ability to provide social support. Resilience and post-traumatic development was found to increase thanks to the shared experiences of paramedics working in a team environment.

An interesting approach to building resilience in paramedics are the use of gamification (Hayes, 2018), virtual reality (Francis et al., 2018), mental health simulation (Pavoni and Story, 2018), and coaching intervention (Janes et al., 2021). Given the nature of their work and the prevalence of mental health, paramedics would benefit from formal training aimed at building resilience (Vaughan et al., 2020). For instance, Somatic Experiencing^®^ (SE^®^) is a resilience-focused trauma treatment designed to address the dysregulation of the autonomic nervous system (ANS) and its impact on physical and mental health (Winblad et al., 2018).

## The Aim of the Study

An overview of scientific databases, such as BASE, EBSCO, PubMed, Medline, Web of Science, and Scopus shows that over 150 articles about the COVID-19 pandemic are published every day. The most scientifically useful ones are based almost exclusively on medical studies. Sadly, few studies thus far have examined the functioning of paramedics during the pandemic. A study by Wasim et al. (2020) showed that 62% of paramedics exhibited symptoms of depression, 65% exhibited symptoms of anxiety, and 55% exhibited symptoms of stress. During the COVID-19 pandemic, paramedics are also at greater risk of stigmatization due to the direct risk of contracting COVID-19 (Zolnikov and Furio, 2020).

The aim of the current study was to establish the role of personal resources (resilience) in the experience of stress by paramedics during the COVID-19 pandemic. Resilience was taken as a measure of personal resources, as during a pandemic situation, strategies of coping with stress are revealed to be insufficient, especially against traumatic stress (Jasielska and Ziarko, 2019).

The following research questions were put forward:

1. Are there differences in stress and resilience levels among paramedics before and during the COVID-19 pandemic?
2. What aspects of resilience lower stress levels among paramedics during the COVID-19 pandemic?

## Materials and Methods

### Participants

In a first phase, before the COVID-19 pandemic (October-November 2019), 75 paramedics (70 men, and 5 women) took part in the study. Their mean age was 38.95 (*SD* = 8.21). They were employed in the emergency response department, hospital emergency department, and medical air rescue, in the Pomorskie voivodeship (Poland).

In a second phase, the research was carried out during the COVID-19 pandemic, from May 10 to June 12, 2020. Eighty-four paramedics (74 men, 10 women) took part in the study. We mention that 84.5% of the participants (71 paramedics—67 men and 4 women) were the same from the pre-pandemic period, that is, from the first phase. Their mean age was 37.46 (*SD* = 7.47). They were employed in the Pomorskie voivodeship, in the same departments as during the pre-pandemic period.

In both situations (pre-pandemic and during COVID-19 pandemic), approximately 67 per cent had a Bachelor’s degree in medical rescue, 25% had a Master’s degree, also in medical rescue, and 10% had post-secondary education in medical rescue.

We used the snowball sampling technique due to the specifics of the investigated participants (being part of hard-to-reach populations). Data were collected using Google forms (Google LLC, Mountain View, CA, United States).

During the pandemic, the paramedics were asked the following question: How many hours per week are you currently working? The mean was 59.42 h (*SD* = 17.09), the median was 60 h, the minimum was 36 h, and the maximum was 110 h. We mention that in the pre-pandemic period the participants average work hours per week were: 56.7 h (*SD* = 17.9). Minimum working hours was 36.0 h, and maximum working hours was 120 h.

The number of confirmed COVID-19 cases remained at a roughly constant level during the study. On the first day of the study, May 10, 345 cases were reported, while on the last day, June 12, this number was 376.

### Measures

The Sense of Stress Questionnaire (Skala Poczucia Stresu) (Makarowski and Plopa, 2010) was used to measure stress levels. It comprises 21 questions, forming three scales: *emotional tension* (7 items, e.g., “I worry that more and more things anger me”), *external stress* (7 items, e.g., “I am criticized too often”), and *intrapsychic stress* (7 items, e.g., “Thinking about my problems makes it difficult for me to fall asleep”). The respondents indicate their responses to each item on a five-point Likert-type scale, from 1 (*not true*) to 5 (*true*). The higher the total score or the scores in the individual scales, the higher the respondent’s stress level. The Sense of Stress Questionnaire has been used in research of, among others, athletes (Makarowski et al., 2020), patients (Wrzeciono et al., 2021), mothers of prematurely born children (Lutkiewicz, 2020), and medical students (Henzel et al., 2016). The scale is characterized by high reliability. The Resilience Scale (*Skala Pomiaru Prężności*, SPP-25) by Ogińska-Bulik and Juczyński (2008) was used to measure resilience. It allows for measuring the general level of resilience, understood as a personality trait, as well as its five factors: *perseverance and determination* (example item: “I try to overcome problems regardless of how difficult they are”), *openness to new experiences and sense of humor* (e.g., “I can notice humor in the situations I encounter”), *competences and tolerance of negative emotions* (e.g., “I can adjust to every situation, even the most difficult ones.”), *tolerance of failure and treating life as a challenge* (e.g., “I easily adapt to new situations”), and *optimism and the ability to mobilize in difficult situations* (e.g., “When faced with difficult situations, I see many solutions”). Each scale is comprised of five items. Respondents give their answers on a s-point Likert-style scale, from 0 (*definitely do not agree*) to 4 (*definitely agree*). The total score is the sum of the five individual factor scores. The higher the score, the higher the respondent’s resilience. The Resilience Scale has been successfully used in research by medical personnel (Ogińska-Bulik and Kobylarczyk, 2015) and among adolescents (Ogińska-Bulik and Michalska, 2019). This scale is very often used to measure resilience and has good psychometric parameters.

### Statistical Analysis

The STATISTICA 13 software was used to analyze intergroup differences and carry out a correlation and regression analysis.

## Results

First, stress and resilience levels before and during the pandemic were compared. The results are shown in Table 1.

**TABLE 1:** Stress and resilience levels among paramedics before and during the COVID-19 pandemic.

**Variable**	**Before the pandemic**			**During a pandemic**			***t***	***p***	**Cohen’s d**
	**ω**	**M**	**SD**	**ω**	**M**	**SD**			
Emotional tension	0.81	17.19	5.49	0.84	15.48	5.59	−1.938	si.	0.31
External stress	0.82	16.04	4.87	0.83	16.14	4.78	0.133	si.	0.02
Intrapsychic stress	0.87	14.24	5.11	0.85	12.24	4.63	−2.586	*	0.41
Perseverance and determination in action	0.80	14.43	3.52	0.80	15.26	3.11	1.587	si.	0.25
Openness to new experiences and sense of humor	0.81	15.37	3.09	0.83	14.50	3.28	−1.723	si.	0.27
Personal coping competences and tolerance of negative emotions	0.88	14.59	3.34	0.86	15.10	3.14	0.989	si.	0.16
Tolerance of failures and perceiving life as a challenge	0.86	13.73	3.23	0.86	15.43	3.25	3.292	**	0.52
Optimistic attitude toward life and capacity for self-mobilization in difficult situations	0.84	18.73	3.23	0.85	13.48	2.72	−11.139	***	1.76

ω, McDonald’s omega; si., statistically insignificant.

*p < 0.05, **p < 0.01, ***p < 0.001.

Paramedics reported higher levels of *intrapsychic stress* before the pandemic. No differences in *emotional tension* and *external stress* were observed.

*Tolerance of failure and treating life as a challenge* was higher during the pandemic, in contrast to *optimism and the ability to mobilize in difficult situations*.

Table 2 shows the results of the correlation analysis (Pearson’s *r*) between the stress and resilience levels before and during the pandemic.

**TABLE 2:** Pearson’s *r* correlations between stress and resilience dimensions among paramedics before and during the COVID-19 pandemic.

**Variable**	**During a pandemic**			**Before the pandemic**		
	**Emotional tension**	**External stress**	**Intrapsychic stress**	**Emotional tension**	**External stress**	**Intrapsychic stress**
Perseverance and determination in action	−0.76***	−0.64**	−0.70***	−0.44***	−0.46***	−0.47***
Openness to new experiences and sense of humor	−0.53***	−0.34***	−0.38***	−0.42***	−0.42***	−0.49***
Personal coping competences and tolerance of negative emotions	−0.74***	−0.61***	−0.71***	−0.48***	−0.51***	−0.58***
Tolerance of failures and perceiving life as a challenge	−0.67***	−0.58***	−0.62***	−0.53***	−0.54***	−0.60***
Optimistic attitude toward life and capacity for self-mobilization in difficult situations	−0.68**	−0.50***	−0.53***	−0.53***	−0.56***	−0.56***

**p < 0.01, ***p < 0.001.

All stress scales were related to the five resilience scales. All correlations were negative and statistically significant. Thus, it can be concluded that the lower the paramedics’ personal resources (resilience), the more stress they experience.

In the second phase of the study, the paramedics were asked if they have contact with patients with COVID-19. The stress and resilience results are shown in Table 3.

**TABLE 3:** Stress and resilience levels among paramedics who had (*N* = 52) and did not have (*N* = 32) contact with patients with COVID-19.

**Variable**	**Contacting COVID-19**				***t***	***p***	**Cohen’s d**
	**Yes**		**No**				
	**M**	**SD**	**M**	**SD**			
Emotional tension	17.39	5.96	13.56	5.28	3.020	**	0.68
External stress	17.82	4.35	14.06	4.92	3.718	***	0.81
Intrapsychic stress	13.68	4.97	10.94	5.00	2.483	**	0.55
Perseverance and determination in action	15.04	3.56	15.31	2.15	−0.399	si.	0.09
Openness to new experiences and sense of humor	14.04	4.25	14.19	2.74	−0.181	si.	0.04
Personal coping competences and tolerance of negative emotions	14.36	4.07	15.56	2.33	−1.537	si.	0.36
Tolerance of failures and perceiving life as a challenge	14.75	3.90	15.94	2.45	−1.553	si.	0.36
Optimistic attitude toward life and capacity for self-mobilization in difficult situations	12.71	3.58	13.94	1.92	−1.791	si.	0.43

si., statistically insignificant.

**p < 0.01, ***p < 0.001.

Statistically significant differences were observed between all stress scales. Paramedics who had contact with COVID-19 patients were characterized by average stress levels (4–5 sten), whereas paramedics who did not have such contact exhibited low stress levels (2–3 sten).

### Predicting Stress Levels

According to the data presented in Table 2, there is a relationship between experienced stress and resilience. In order to identify those aspects of resilience which allow for predicting stress levels, a regression analysis was carried out. Data distributions met the normality assumption (regression standardized residual are normally distributed, Shapiro-Wilk test > 0.05). Also, we underline that the correlations between the independent variables (IVs) are not high (Pearson correlation values < 0.50), and multicollinearity was being avoided (Leech et al., 2005). Because the *Std. Residual* and the *Stud. Residual* scores are not comprised in the [−3, +3] interval, and the Cook’s Distance values are less than 1 (Field, 2000), no extreme or influential cases were noticed, which otherwise could influence the values of the regression coefficients. Considering homoscedasticity, the residuals form a cloud of points randomly (the scatterplots of the residuals were analyzed).

A backward stepwise regression was carried out.

The three stress dimensions—*emotional tension, external stress*, and *intrapsychic stress*—were the dependent variables. The five aspects of resilience were the independent variables. Table 4 shows the regression coefficients of the statistically significant predictors.

**TABLE 4:** Multiple regression analysis results with stress dimensions as the dependent variable and resilience during the COVID-19 pandemic as the independent variable.

**Dependent variable**	**Independent variables**	**β**	**B**	**SE B**	***t***	***p***
Emotional tension	Constant term		36.79	1.84	19.970	***
	Perseverance and determination in action	−0.32	−0.53	0.20	−2.687	**
	Openness to new experiences and sense of humor	−0.58	−0.82	0.16	−4.958	***
	*R*^2^ = 0.73; *F*(2.53) = 71.93***; *ME* = 3.15					
External stress	Constant term		30.35	1.88	16.187	***
	Perseverance and determination in action	−0.68	−0.83	0.12	−6.865	***
	*R*^2^ = 0.54; *F*(1.54) = 47.122***; *ME* = 3.27					
Intrapsychic stress	Constant term		27.76	1.48	18.717	***
	Personal coping competences and tolerance of negative emotions	−0.80	−0.98	0.10	−9.862	***
	*R*^2^ = 0.63; *F*(1.54) = 97.25***; *ME* = 2.99					

**p < 0.01, ***p < 0.001.

*ME, mean error.*

### Emotional Tension

The regression analysis shows that *emotional tension* among paramedics working during the pandemic is determined by two significant independent variables. As *perseverance and determination* and *openness to new experiences and sense of humor* increase, *emotional tension* decreases (negative beta coefficients). The second factor had a greater predictive role. The corrected determination coefficient, which is a measure of model fit, indicated that 73% of the variance in *emotional tension* can be explained by two statistically significant independent variables entered into the regression equation.

### External Stress

In the second regression equation, where *external stress* was the explained variable, a negative beta coefficient occurred for *perseverance and determination.* This variable was the only significant predictor of *external stress*. The more *perseverance and determination*, the lower the *external stress* levels. The determination coefficient was *R*^2^ = 0.54.

### Intrapsychic Stress

A multiple linear regression analysis showed that the model predicted 63% of the variance in one variable—*competences and tolerance of negative emotions* (negative beta coefficient). It can be concluded that *intrapsychic stress* increases as *competences and tolerance of negative emotions* decrease.

## Discussion of Results

The COVID-19 pandemic has impacted global functioning. Most countries had to confront its consequences to a greater or lesser extent. A rapid increase in patient numbers, insufficient equipment stocks and personnel numbers, and the shrinking capacities of the medical sector have put significant strains on medical personnel. For paramedics, overwork and concern for own health, as well as the health of one’s significant others, have joined the standard workplace stressors of hurry, shift work, and responsibility for the patients. In such a situation, the paramedics’ psychological condition becomes even more important for their effectiveness. The current study attempted to answer the following questions: What were the stress levels among the paramedics before the pandemic, and what are they during the pandemic? Which aspects of the personal resource of resilience lower the stress levels experienced by paramedics during the pandemic?

The results revealed that *intrapsychic stress* is lower than in the pre-pandemic period. On the other hand, there were no differences in *emotional stress* and *external stress*. We can assert that the differences shown for *intrapsychic stress* are due to the COVID-19 pandemic (and not to individual differences), taking into account that almost 85% of the participants where the same individuals, tested in the two phases of the research (pre-pandemic and during the pandemic). It can be expected that, during the COVID-19 pandemic, stress levels would increase rather than decrease. These results can be interpreted in several ways.

First, activating of personal resources—resilience. In difficult situations, people activate their personal resources (including resilience), which might stimulate their development. An analysis by Labrague (2020) shows that medical personnel exhibits medium to high resilience levels during the pandemic. Also, in light of the first corollary which follows from the Conservation of resources (COR) theory’ basic principles (Hobfoll, 1989; Halbesleben et al., 2014), people having greater resources are less vulnerable to negative stress following traumatic or chronic stress (Holmgreen et al., 2017). Together with the medium or high resilience, paramedics had more resources to begin with, including privileged social status (e.g., most being men, white race, and having a good income), and cultural capital—comprising education, intellect, skills, etc. But activating personal resources (such as resilience) can be exhausting for paramedics, which can lead to increased stress levels in the long term (a demanding job can erode health). As Linton et al.’s (2015) review highlighted, high job demands predict future sleep difficulties/disturbances. Also, work stress, alongside organizational climate predict the emotional exhaustion of employees and are linked with depressive symptoms (Gayman and Bradley, 2013). The current study was carried out in May and June 2020, at the beginning of the pandemic, when the level of reported infections was relatively low. Thus, the personal resource of resilience might not have become depleted yet. If facing this depletion, replenishment can take place (sleep being an essential component in this dynamic) or depletion can spiral downwards, with negative consequences on work-related outcomes (Barnes, 2012). In this context, we also mention Österberg et al. (2014) findings, according to which, despite considerable recovery, employees with previous work-stress-related exhaustion manifested important signs of a minor attention deficit when returning to work.

Second, habituation—loss of reaction to repeated stimuli. Reactions decrease, and, in time, can disappear, as a result of repeated exposures to a constant stimulus (Wochyński et al., 2020). Habituation plays an adaptive function, as it allows for an effective utilization of emotional and cognitive resources.

Third—the COVID-19 pandemic has placed appropriately high priority on the basic issues of health and life. The threat of infection with the coronavirus, in a situation of rigorous following of health and social isolation protocols, was low during the period in which the study was carried out. On the other hand, in the face of a global pandemic, other stressors are unable to lower one’s mood. If such fundamental values and health and life are at risk, people judge everyday problems differently (Ogińska-Bulik and Juczyński, 2016).

In the second phase of the study, the paramedics were asked if they had contact with patients with COVID-19 during their work. In the sample of 84 paramedics, 52 (62%) had such contact. A comparison of these groups showed that they were characterized by higher stress levels on each dimension (*emotional tension, external stress*, and *intrapsychic stress*). However, it must be noted that those paramedics who had contact with patients with COVID-19 exhibited medium stress levels, whereas other paramedics exhibited low stress. Experiencing stress can increase a sense of threat to one’s health and life (Stasiła-Sieradzka and Turska, 2019).

Both paramedics who had and who did not have contact with patients with COVID-19 were characterized by similar resilience levels. Resilience was revealed to be negatively correlated with stress on a moderate level. As resilience increases, experienced stress decreases (Ogińska-Bulik and Michalska, 2019). In order to examine the effect of resilience on stress, a regression analysis was carried out with the three dimensions of stress considered in the current study as the dependent (explanatory) variables and with the five dimensions of resilience as the independent variables. The analysis was intended to indicate which of the aspects of resilience decrease or increase the experienced stress among paramedics during the pandemic. However, it must be noted that the regression analysis only included those paramedics who had contact with patients with COVID-19. Those paramedics who did not have such contact were not included, as they constituted too small a sample.

The analysis of the predictive power of the individual aspects of resilience for the subjective experience of stress revealed that *perseverance and determination* played a key preventive role. This factor was a statistically significant explanatory variable in both regression equations. A study on grieving people showed that *perseverance and determination* was the main predictor of positive post-traumatic changes (Felcyn-Koczewska and Ogińska-Bulik, 2012). This suggests that different aspects of resilience may have a significant influence on post-traumatic growth, and this, in turn, depends on its kind, among others. *Perseverance and determination* were the most significant for preventing increases in experienced stress. It can be expected that experiencing stress at work every day builds immunity. Negative consequences of stress can coexist with its positive effects. Effective coping with difficult situations builds personal resilience, which makes individual cope more effectively with future difficulties. A study by Janoff-Bulman (2004) shows that earlier coping with traumatic experiences builds and strengthens personal resources, including resilience. This may cause individuals to cope more effectively with similar situations in the future. Paramedics are expected to function effectively in traumatic situations.

Paramedics experience *emotional tension* and *external stress* every day. Only those with appropriate (low-reactivity) personality structures can work as paramedics without incurring excessive costs. *Perseverance and determination* are important for paramedics, and are shaped with experience. Paramedics never know the results of their actions in advance. Sometimes, lives can be saved even in a hopeless situation. Therefore, paramedics are expected to persevere until the end and to not express doubts, as they are responsible for their patients’ lives.

Paramedics who did not have contact with patients with COVID-19 exhibited low stress levels. On the other hand, paramedics who did have such contact were characterized by medium stress levels. This begs the question why their stress levels were medium instead of high. According to Hobfoll’s (1989) theory, stress occurs when (a) personal resources have been diminished, (b) there exists a likelihood, that they may become diminished, or (c) the invested resources are not yielding the expected gains. The extent of the experienced stress depends on the initial levels of personal resources (Chen et al., 2015). It is likely that, among paramedics, these were high. This may have led them to only use a part of their resources during the pandemic situation. This would result in average rather than high stress levels during the pandemic.

The results shown in Table 1 allow for hypothesizing that the COVID-19 pandemic has activated defense mechanisms, which prevented increases in *emotional tension* and *external stress*. *Intrapsychic stress* levels were even lower than before the pandemic. These mechanisms have caused *tolerance of failure and treating life as a challenge* to be higher during the pandemic. On the other hand, these mechanisms did not prevent *optimism and the ability to mobilize in difficult situations* from significantly decreasing during the pandemic.

Resilience, conceptualized as a self-regulation mechanism, is universal and should protect from the negative consequences of both traumatic experiences as well as everyday stressors. In the first model (regression equation), *openness to new experiences and sense of humor* largely determined *emotional stress* levels. So-called isolation is a defense mechanism frequently used by paramedics to cope with extreme situations. Humor and jokes allow for isolating oneself from such situations. Such reactions lower the tension in the emergency rescue team. Humor increases individual coping skills in the face of workplace stress through a shift in perspective, distancing from problems, and a change in the perception of stressors as less threatening (Kruczek and Basińska, 2018).

In the third regression equation, where the explained variable was *intrapsychic stress*, *competences, and tolerance of negative emotions* was the only significant predictor. *Intrapsychic stress* appears together with a sense of difficulty in realizing one’s tasks and overcoming everyday challenges, and of insufficient personal resources. People exhibiting high *intrapsychic stress* experience tension over accepting their problems and exhibit anxiety and withdrawal tendencies when thinking about their future. Such people blame themselves for their problems and they do not consider the role of the social environment. Professional competences are a resource which can lower this type of stress. Having competences necessary to fulfill given professional duties lowers the sense of overwork (Jurek, 2019). The second element of the predictors in the equation were *competences and tolerance of negative emotions.* The ability to cope and tolerate difficult emotions allows effective functioning in stressful situations (Fergus, 2018).

## Conclusion

The results of the current study add to the understanding of the relationship between stress caused by the difficult COVID-19 pandemic situation and psychological resilience. Due to possessing the personal resource of resilience, paramedics are able to effectively cope with stress. Paramedics who have contact with patients with COVID-19 experienced higher stress. Activating personal resources in a long-term pandemic situation can lead to their depletion. Even if the sample of participants is not too large to generalize results (the representativeness of the sample could be seen as a limit of our research), it is important to monitor paramedics’ psychological condition also after the pandemic ends. This occupational group, together with hospital staff, are at the greatest risk of infection with the coronavirus. Another limitations of the research can be drawn. The results could be different in other settings, e.g., if paramedics from other countries, differently affected by the pandemic, would be investigated, or paramedics belonging to different social groups—knowing that being female, elderly and having a racial minority status, together with resource loss, represent predictors of psychological distress (Adeola, 2009; Paul et al., 2014).

Our research conclusions offer valuable resources for medical staff and psychologists (and not only these occupational groups), raising awareness of how stress levels tend to vary in a situation, such as a pandemic—in certain social categories—raising awareness, also, regarding the importance of developing personal resources in people, in order to maintain a “healthy stress” at work (on long-term). Even more during a pandemic, active managerial (see Harkness et al., 2005; Ganster et al., 2011; Wan, 2013) and psychological actions (effective means of coping with work stress) come to support employees (including paramedics), to prevent and reduce, on long run, “unhealthy stress.” However, as pointed out by Janes et al. (2021), the importance of building not only personal but also organizational and systemic resilience of front-line health professionals should be emphasized.

Considering the existing gap in the literature, our study results can represent a starting point for future researchers, eager to better understand the resilience and stress levels of paramedics, working in various settings.

## Data Availability Statement

The raw data supporting the conclusions of this article will be made available by the authors, without undue reservation.

## Ethics Statement

Ethical approval was not provided for this study on human participants because this study was carried out in accordance with the recommendations of the APA and Code of Ethics and Professional Psychologist of the Polish Psychological Association (PTP). The patients/participants provided their written informed consent to participate in this study.

## Author Contributions

All authors listed have made a substantial, direct and intellectual contribution to the work, and approved it for publication.

## Conflict of Interest

The authors declare that the research was conducted in the absence of any commercial or financial relationships that could be construed as a potential conflict of interest.
